# Atomic Layer Etching of Nickel Using N_2_/H_2_ Plasma Exposure and Hexafluoroacetylacetone

**DOI:** 10.1021/acsaelm.5c02655

**Published:** 2026-04-24

**Authors:** Ali Mohamed Ali, Guillaume Krieger, Jean-Philippe Soulié, Harm C. M. Knoops, Wilhelmus M. M. Kessels, Stefan De Gendt, Souvik Kundu, Jean-François de Marneffe

**Affiliations:** 1 Dept. of Chemistry, K.U. Leuven, Celestijnenlaan 200F, Leuven B-3001, Belgium; 2 imec v.z.w., Kapeldreef 75, Leuven B-3001, Belgium; 3 Dept. of Applied Physics, Eindhoven University of Technology, P.O. Box 513, Eindhoven 5600 MB, Netherlands; 4 Oxford Instruments Plasma Technology, Govier Way, Bristol BS35 4GG, United Kingdom

**Keywords:** atomic layer etching, nickel, plasma-enhanced etching, N_2_/H_2_ plasma, Hhfac, anisotropic etching, extreme ultraviolet lithography

## Abstract

Nickel (Ni) and its aluminides are key materials in extreme ultraviolet lithography masks and nanoscale interconnects, where precise patterning is essential. However, the engineering of Ni-based intermetallics poses significant challenges due to their high physical stability and chemical inertness. This study introduces a plasma-enhanced atomic layer etching (ALE) method for Ni, relying on a surface modification by a N_2_/H_2_ plasma mixture followed by selective removal of the modified layer with hexafluoroacetylacetone vapor. Optimizing plasma chemistry, power, and exposure time promotes a controlled surface modification, which minimizes surface roughness and enhances process control. Half-reactions are shown to be self-limited, leading to an etch per cycle of 0.21 ± 0.03 nm at 350 °C. Periodic O_2_ plasma steps are incorporated to eliminate carbon residues from the surface. X-ray photoelectron spectroscopy reveals a mechanism involving surface nitridation and subsequent removal of the Ni_*x*_N layer. The ALE process is demonstrated on blanket substrates and assessed on prepatterned 3D nanostructures to examine the etching directionality. Transmission electron microscopy studies conducted on the blanket and 3D-structured Ni demonstrate the damage-free characteristics and anisotropic nature of the ALE process. The proposed method represents a significant advance in ALE technology and paves the way for anisotropic Ni patterning, which is essential for the fabrication of future nanoscale devices.

## Graphical abstract

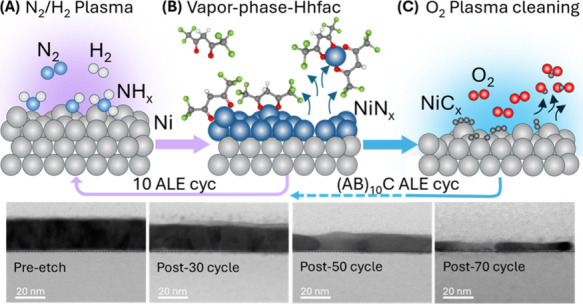

## 1 Introduction

Nickel (Ni) is a highly versatile element with a wide range of applications, valued for its unique properties such as corrosion resistance, chemical stability, mechanical hardness, and economic affordability.^1,2^ Nickel is considered as a novel mask absorber in extreme ultraviolet (EUV) photolithography due to its high extinction coefficient (*k*) at the wavelength of 13.5 nm.^3^ This property allows for the creation of thinner mask absorber layers, thereby reducing shadowing effects and improving the performance of the EUV photolithographic processes. Nickel, as a composite or in Permalloy form (NiFe), is also considered as an electromagnetic shield for RF applications.^4,5^ Ni_*x*_Al_*y*_ alloys are investigated as alternative EUV absorbers, but also as potential replacements for copper interconnects in microelectronics, particularly at the smallest line widths, where traditional materials show too high resistivity.^6^ Nickel and alloys such as NiFeSi can be used as magnetostrictive layers for nanodevices based on surface acoustic waves.^7–9^ Finally, Ni is considered as a potential metal contact to transition-metal dichalcogenides, in the stacked nanosheets geometry envisioned to replace silicon from the 2 Å technology node.^10^ All of the applications cited above require, during their fabrication, the etching, anisotropic or isotropic, of elemental nickel or nickel-containing alloys.

A key challenge in the etching of Ni-based structures stems from the low volatility of Ni-halide compounds.^11,12^ The two standard techniques for metal etching are (i) ion beam milling (IBM) and (ii) reactive ion etching (RIE). Both methods have significant limitations when applied to Ni. IBM struggles with poor selectivity and reduced etch rate in high aspect ratio features.^13^ It also causes considerable damage to the remaining layers and leaves behind metal/oxide residues that require complex cleanup processes.^14^ RIE, a plasma-based technique widely used for metal etching, relies on chemical reactions between reactive halogen radicals (F, Cl, and Br) and the surface. However, it is less suited for Ni, due to nickel’s high chemical inertness, which resists the formation of volatile compounds with halogen etching gases.^15^ As a result, innovative approaches are needed to achieve the efficient nanoscale etching of Ni.

Recently, some new methods were proposed, based on chelating chemistry, i.e., ligand-exchange or ligand-addition reactions,^16,17^ aiming at forming volatile metal–organic compounds. However, the inherent low reactivity between organic ligands and metallic Ni, or dissociation of the organic molecules on the Ni surface, presents challenges in forming organo-metallic compounds. Therefore, presurface modification is necessary to localize electrons and promote the formation of organo-metallic bonds.

Previously, the atomic layer etching (ALE) of Ni was enabled by modifying the surface with an oxygen plasma, followed by removal of the NiO_*x*_ layer using chelation with acetic acid solution or formic acid vapor.^18^ A similar approach combines plasma nitridation with formic acid vapor.^19^ Formic acid decomposition can lead to gradual surface contamination over time, as the N_2_ plasma generates mainly N radicals, which may etch carbon slowly but are not effective in forming volatile carbon compounds. This contamination necessitates an additional Ar^+^ ion beam sputtering (IBM) step between cycles to remove accumulated residues. However, this additional IBM cleaning step might fall short for high aspect ratio structures.

To overcome these limitations, there is growing interest in clean ALE processes that do not generate heavy residues. N_2_- and H_2_-based plasmas have emerged as effective alternatives, offering both surface modification and sustainable residue removal, with demonstrated benefits in etch rate, selectivity, and surface quality across various plasma etching processes.^20^ For instance, incorporating moderate N_2_ levels into a hydrogen-based plasma significantly enhances copper etching, boosting both etch rates and selectivity between Cu and SiO_2_.^21^ Also, N_2_/H_2_ plasma in atomic layer etching of low-k materials leads to an ion-enhanced etch process with CN sidewall passivation, giving the desired anisotropic shape and uniform etch rate.^22^ By maintaining surface integrity throughout the etching process, N_2_/H_2_ plasmas may offer a viable alternative to conventional ALE methods, achieving lower contamination levels and reduced process complexity.

Among organic etchants, only a few have demonstrated effectiveness in Ni etching. The feasibility of using a hexafluoroacetylacetone (Hhfac) for Ni etching has been demonstrated theoretically. Basher et al. found that the Hhfac anion (hfacβ) bonds in a stable manner with positively charged Ni atoms on NiO_*x*_ surfaces, making it a suitable candidate for thermal ALE of Ni.^23^ In addition, Hhfac exhibits easy adsorption onto nickel oxide surfaces without an energy barrier, whereas it has a tendency to decompose on metallic Ni surfaces,^24^ providing NiO_*x*_:Ni selectivity and allowing for self-limiting surface reactions, which are essential for ALE. While theoretical studies demonstrate the feasibility of using Hhfac for Ni etching, there are challenges in translating these findings into experimental practice. For instance, Sang et al. highlighted that Hhfac lacks efficacy in etching NiO,^25^ underscoring potential gaps between theoretical predictions and practical outcomes.

The present study proposes, for the first time, an atomic layer etching (ALE) process for nickel (Ni) utilizing a N_2_/H_2_ plasma mixture for surface modification combined with a hexafluoroacetylacetone (Hhfac) chelating agent for the removal of the modified surface layer. The process leverages the benefits of N_2_/H_2_ plasmas for enhancing the selectivity and anisotropy, improving surface quality, and minimizing contamination. Periodic O_2_ plasma steps are integrated to promote the efficient removal of surface-bound carbon. This approach establishes a practical and precisely controlled ALE method for Ni.

## 2 Experimental Procedures

### Ni Film Deposition

The Ni films were deposited on blanket and patterned wafers using a 300 mm physical vapor deposition (PVD) system by Canon Anelva. The deposition was conducted at room temperature with a 6 in. pure Ni target, utilizing a 300 W DC Ar plasma discharge. A uniform (unpatterned) 20 nm-thick Ni film was deposited on a 100 nm-thick SiO_2_ on a Si blanket wafer. 3D structures were fabricated using imec’s 300 mm pilot line with a shallow trench isolation (STI) process. Si wafers were oxidized to form a 5 nm SiO_2_ pad oxide, followed by a 50 nm Si_3_N_4_ deposition. A double sacrificial bilayer hardmask (SoC/SoG) was applied and patterned using 193 nm immersion lithography to create a relaxed step-like structure. Etching was performed in a 300 mm plasma chamber (Lam Kiyo) through multiple steps to remove the bilayer hardmask, Si_3_N_4_, pad oxide, and bulk silicon by using an HBr/Cl_2_-based process. Residual SoC was removed in situ with a N_2_–O_2_ plasma, and the Si_3_N_4_ hardmask was stripped using a selective wet etch process, leading to 300 nm deep structures into bulk Si, with variable horizontal spacings, forming trenches (100 nm, isolated, or dense) to isolated lines (100 nm) and steps. Then, a Ni film was deposited under the same conditions as the blanket film, with wafer tilt and rotation applied to ensure conformal coverage over the 3D structure. After deposition, the wafers were cleaved into smaller coupons to perform the atomic layer etching (ALE) process.

### ALE Process of Ni

The ALE process for Ni was conducted in an Oxford Instruments PlasmaPro ASP reactor,^26^ equipped with a remote capacitively coupled plasma (CCP) source operating at 13.56 MHz, a 200 mm substrate table with a temperature controller, a turbomolecular pump, and a load lock. The as-deposited Ni films were etched by using an ALE cyclic process consisting of two steps. The surface modification step was performed using different plasma chemistries as shown in Table 1, followed by a removal step where the modified metal surface was exposed to hexafluoroacetylacetone (Hhfac) organic vapor. All plasma exposures were performed at a power of 100 W, a pressure of ∼350 mTorr, and were followed by a purge and a pump step. For the N_2_/H_2_ plasma, different power of 100–300 W was supplied to the plasma source, and various H_2_/(H_2_ + N_2_) ratios were used with a total flow rate of 120 sccm. Hhfac (≥99% ReagentPlus; CAS 123–54–6) from Sigma-Aldrich was employed for the surface removal step. Hhfac was maintained at 15 °C in a stainless-steel container with a Peltier cooling system. Hhfac was vapor drawn into the chamber in multiple pulses of 0.5 s each, followed by a 5 s hold step at ∼1000 mTorr, with intermittent Ar purges (300 sccm) lasting 2 s. After every 10 ALE cycles, the Ni surface was cleaned with 30 s of O_2_ plasma at 100 W. All ALE processes were carried out at a table temperature of 350 °C unless specified.

**Table 1:** ALE Process Conditions with Different Plasma Chemistries

	ALE process sequence		
conditions	A	B	C
i	O_2_ plasma	Hhfac	
ii	O_2_ plasma	Hhfac	H_2_ plasma
iii	N_2_ plasma	Hhfac	
iv	N_2_ plasma	Hhfac	H_2_ plasma
v	N_2_/H_2_ plasma	Hhfac	

### Analytical Methods

*Ex situ* X-ray reflectivity (XRR) measurements were carried out to determine the thickness reduction post-ALE process. The uncertainty of the XRR fitting, evaluated by the figure of merit, was within ± 0.2, and the extracted film thicknesses were in good agreement with the cross-sectional TEM measurements. Crystallinity pre- and postetching was examined by grazing incidence X-ray diffraction (GIXRD) scans. Both XRR and GIXRD measurements were conducted on the Bruker D8 Advance diffractometer with parallel-beam optics, Gobel mirror, using Cu Kα radiation (λ = 1.54Å) generated at 40 kV and 40. X-ray photoelectron spectroscopy (XPS) measurements were carried out using a Thermo Scientific KA1066 spectrometer equipped with a monochromatic Al Kα X-ray source (hν = 1486.6 eV) to conduct a first-pass analysis of the film after the ALE process using various conditions. The resulting XPS spectra were calibrated to the C 1s reference peak at 284.9 eV and fitted using CasaXPS. Depth profiling was performed by Ar^+^ ion sputtering at an ion energy of 500 eV for 3s. The surface morphology of the etched films was studied by performing atomic force microscopy (AFM) measurements using a Bruker Dimension Icon. Transmission electron microscopy (TEM) cross-section analysis was performed after preparation of a lamella using dual beam FIB/SEM Helios450HP. These measurements were also used to corroborate the X-ray reflectivity data.

## 3 Results and Discussion

### 3.1 Influence of Plasma Chemistry, Power, and Exposure Time

Various plasma chemistries were initially screened to establish the most effective surface modification step, as summarized in Table 1. The etch per cycle (EPC) values of Ni under five different process sequences are summarized in Figure 1(a). Etching experiments were performed on blanket Ni films with the etch rate monitored after 30 cycles using X-ray reflectometry (XRR). The corresponding XPS Ni 2p_3/2_ and F 1s fitted spectra of Ni surface after etching, as well as depth profile XPS of Ni 2p_3/2_, F 1s, O 1s, N 1s, and C 1s are shown in Figure 1(b) and (c), respectively. Curve fitting of Ni 2p_3/2_ and F 1s spectra was carried out using a mixed Gaussian/Lorentzian function after background subtraction by the Shirley method.^27^ The Ni 2p_3/2_ spectra were deconvoluted into a main nickel peak (Ni-M), a shoulder peak associated with surface-effect contributions related to oxidation or fluorination (NiO_*y*_F_*z*_), and a satellite peak at 853, 855–858.5, and 860–864.5, respectively.^28^ The F 1s spectra were resolved into two dominant components, which are attributed to nickel fluorination (658.5 eV) and organic fluoride species (688.4).

**Figure 1:**
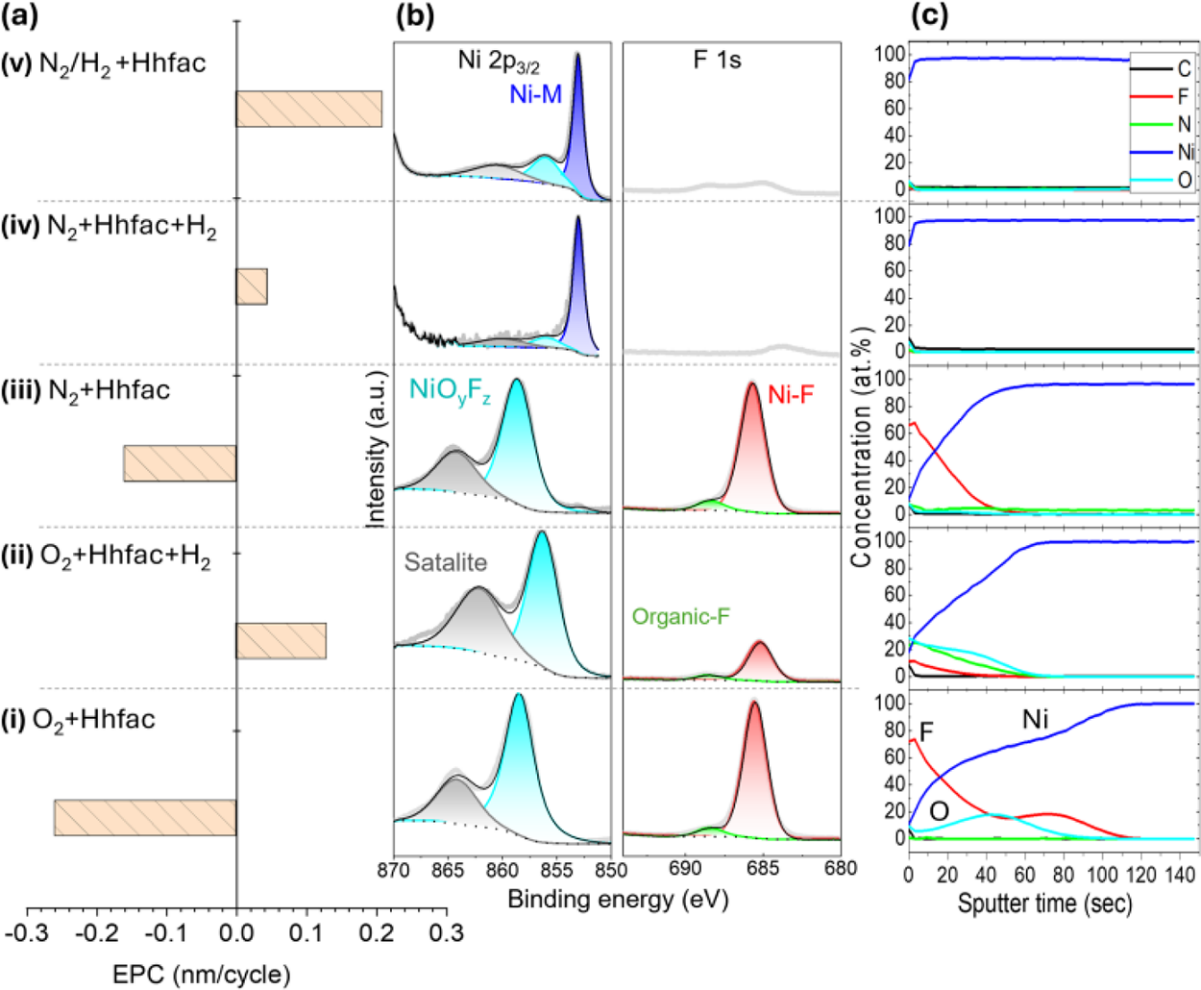
(a) Etch per cycle of nickel using various plasma exposures (labeled (i) through (v)) and Hhfac dosing, (b) related XPS surface scans of Ni 2p_3/2_ and F 1s regions, and (c) depth profile XPS including C, F, N, Ni, and O signals.

For processes using pure O_2_ or N_2_ plasma with Hhfac (conditions i and (iii), an increase in film thickness was observed instead of etching. This anomalous thickening is accompanied by a prominent peak in the XPS F 1s spectra at a binding energy (BE) of 685.5 eV, alongside the NiO_*y*_F_*z*_ in the XPS Ni 2p_3/2_ spectra shifted to a higher binding energy (858.5 eV).^29^ These peak positions indicate the formation of a nickel fluoride-based material (NiF_2_). Furthermore, depth profiling confirms the formation of a thick NiF_2_-rich overlayer, along with oxygen incorporation, suggesting complex surface chemistry involving nickel, fluorine, and oxygen. The observed accumulated material is likely because Hhfac molecules tend to dissociate on the surfaces,^23^ releasing fluorine, which reacts with nickel to form stable, nonvolatile NiF_2_, or leaves over some organic fluoride (C–F) residuals. This reaction pathway promotes material buildup rather than layer removal, resulting in a net increase in the film thickness.

To overcome this limitation, H_2_ plasma was introduced into the ALE process sequence. In the ABC ALE sequence (condition (ii) of the O_2_ plasma, Hhfac, and H_2_ plasma, an EPC of 0.13 nm/cycle was achieved. A downward shift of the NiO_*y*_F_*z*_ peak to 855 eV was observed, suggesting the H radicals may inhibit the formation of NiF_2_ by suppressing fluorine incorporation on the Ni surface. Nevertheless, the XPS depth profile revealed residual oxygen on the surface, indicating incomplete removal of reaction byproducts and suboptimal ALE performance. The ABC sequence (condition (iv) of N_2_ plasma, Hhfac, and H_2_ plasma shows barely measurable etching but results in a surface free of fluorine impurities, suggesting that H radicals can effectively suppress Ni fluorination even though the overall etch efficiency remained low.

The most promising outcome was obtained using N_2_/H_2_ plasma followed by Hhfac dosing (condition (v)) with an EPC of 0.21 nm/cycle. XPS analysis detected faintly visible NiF_2_-related peaks, pointing to a cleaner surface and more efficient material removal. These insights indicate that the simultaneous presence of H and N radicals in N_2_/H_2_ plasma plays a key role in mitigating Ni fluorination and enhancing etch performance.^30^ Accordingly, the results demonstrate that while O_2_ and N_2_ plasmas followed by Hhfac tend to promote NiF_2_ growth, the incorporation of H_2_, particularly mixed in N_2_/H_2_ plasma, shifts the chemistry toward effective ALE by suppressing fluorination and enabling higher EPC values.

The dependence of the etch rate on the mixing ratio between N_2_ and H_2_ gases in the plasma was further investigated. ALE cycles were conducted using alternating pulses of plasma with varying H_2_/(H_2_ + N_2_) ratios, followed by Hhfac dosing, as shown in Figure 2a. The N_2_/H_2_ plasma was conducted at source power and exposure times of 100 W and 5 s, respectively. The EPC exhibits a strong dependence on the H_2_ fraction, with a maximum EPC observed at H_2_/(H_2_ + N_2_) = 0.67. This maximum might have several causes, such as a difference in radical density or the generation of NH_*x*_ species.^31^ The EPC trend shows consistency with the plasma-activated catalytic generation of ammonia, where optimizing atomic N and H fluxes in N_2_/H_2_ plasmas can efficiently generate ammonia with more than 10% of the total background pressure.^32,33^ In addition, a catalytic surface like Cu, Ni, or other 3d transition metals effectively catalyzes ammonia production from N_2_ and H_2_ in nonthermal atmospheric plasma at a ratio of H_2_/N_2_ = 3.^34,35^ A DFT study on the adsorption of ammonia on Ni surface has reported that NH_3_ can donate electrons to surface Ni atoms, slightly relax the topmost layer, and lower the work function by 1–2 eV.^36–38^ Thus, the relaxation effect weakens pre-existing metal bonds, lowering the activation barrier and could therefore help to enable facile exchange with incoming β-diketonate ligands such as Hhfac.^39^

**Figure 2:**
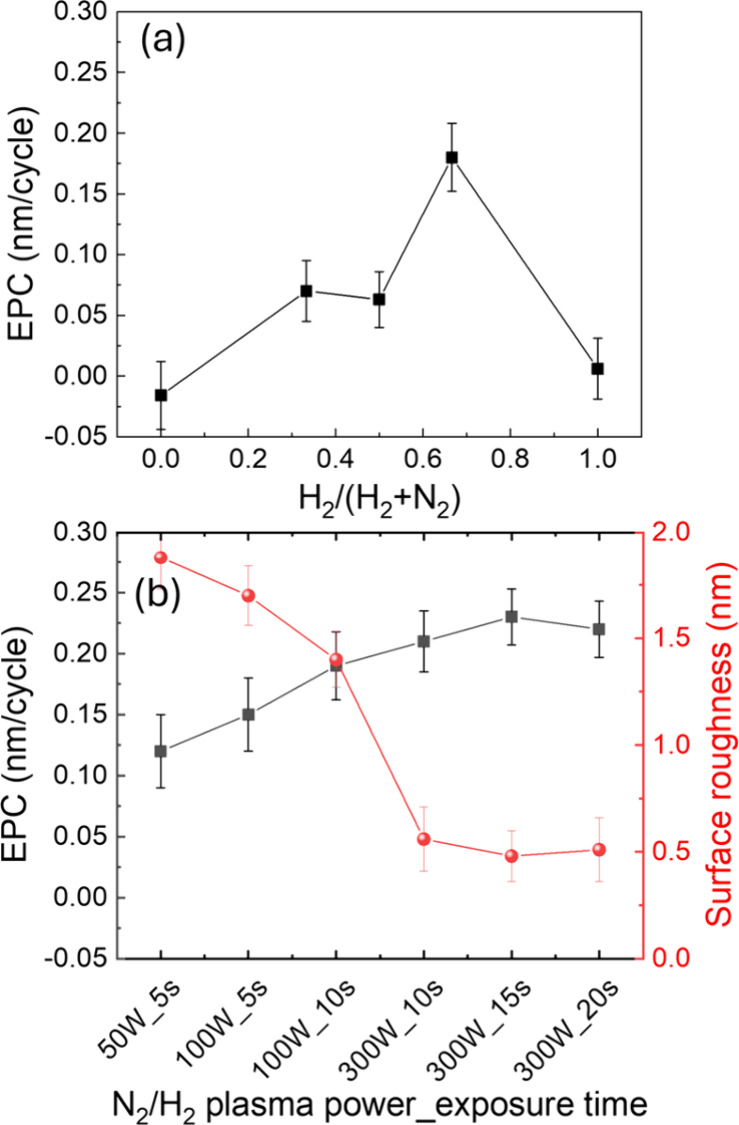
(a) Etch per cycle (EPC) for Ni as a function of the mixing ratio between N_2_ and H_2_ gases in the plasma. (b) EPC in black and surface roughness in red of Ni etched at H_2_/(H_2_ + N_2_) = 0.67 with different combinations of plasma power and exposure time.

The etch rate and postetch surface morphology were investigated as a function of N_2_/H_2_ plasma power and exposure time, under a fixed H_2_/(H_2_ + N_2_) ratio of 0.67. Figure 2b shows the EPC and surface roughness as functions of the plasma power (50–300 W) and exposure time (5–20 s). The EPC increases with plasma power and time, which could be explained by the increasing ion and radical flux incident on the surface,^40^ and a maximum EPC of 0.21 nm/cycle was achieved with a power of 300 W for 15 s. Notably, the higher plasma power not only increases the EPC but also leads to a smoother postetch surface compared to lower power plasma.

Figure 3 shows AFM images of the Ni surface before and after etching, for different plasma conditions. At low powers of 50–100 W, Figure 3b,c, the etched Ni film shows a higher root-mean-square (RMS) roughness of 1.8 nm (50 W, 5 s) and 1.7 nm (100 W, 5 s) compared to the pristine Ni film (0.35 nm RMS). The roughened morphology is characterized by the appearance of grooves and contour-like features, suggesting preferential etching at specific surface sites. To further examine the origin of this surface roughening, cross-sectional TEM analysis was performed on a Ni film etched at a plasma power of 100 W for 5 s after 60 and 100 ALE cycles. As shown in Figure 3e,f, the TEM images reveal localized etching along some kind of grain boundary features, indicated by yellow arrows, that are in line with the AFM observations. It suggests that grain boundaries or more reactive crystalline grain orientations are preferentially etched under lower plasma power conditions.^41^ Preferential adsorption of hydrogen-derived intermediates at grain boundaries has been reported in Ni and related transition-metal systems, where grain boundaries stabilize active hydrogen or NH_*x*_ species and modulate local reactivity.^42–44^

**Figure 3:**
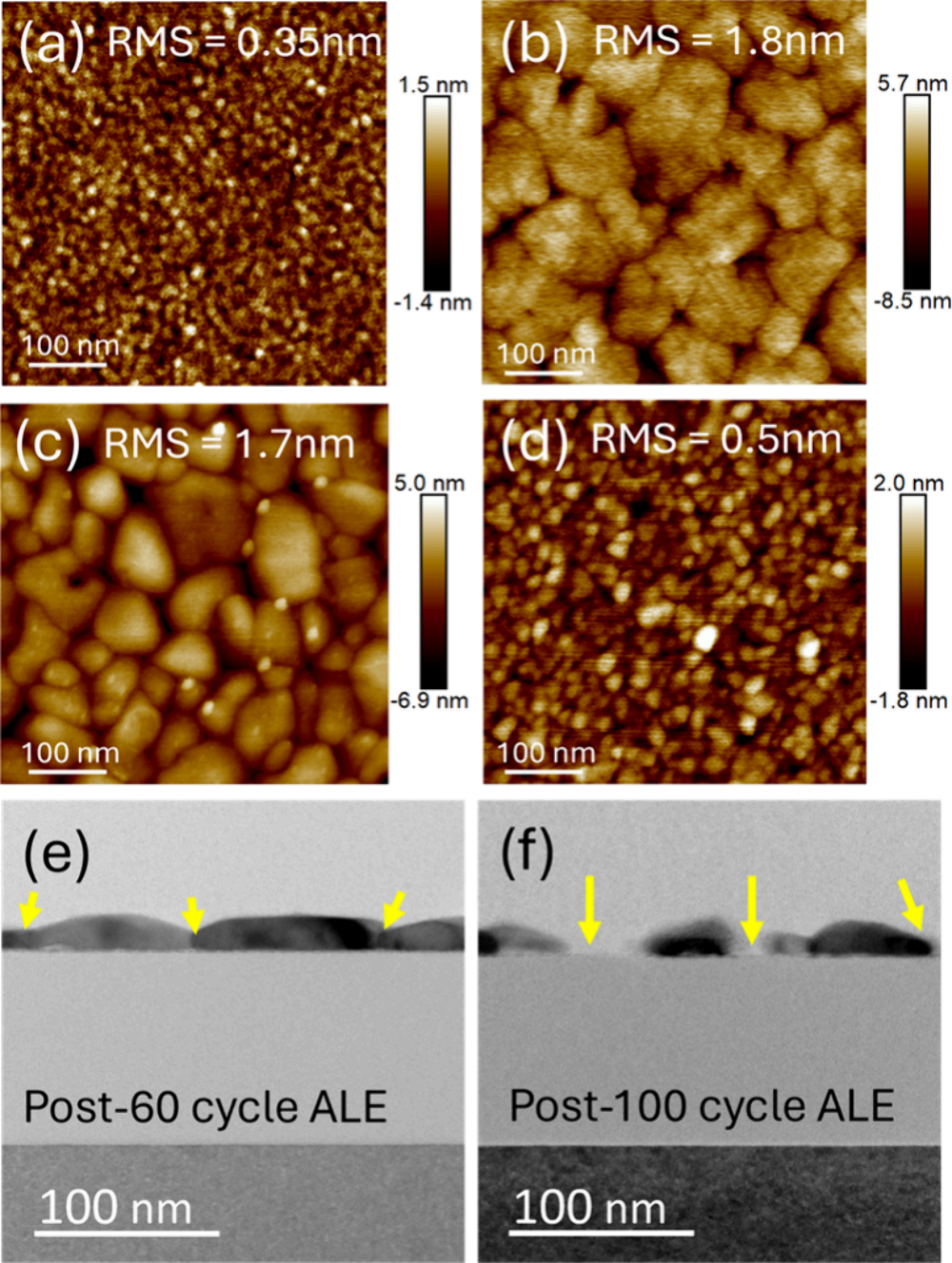
AFM scan of a 500 × 500 nm^2^ region of Ni (a) pristine, and postetch at different N_2_/H_2_ plasma powers and exposure times of (b) 50 W for 5 s, (c) 100 W for 5 s, and (d) 300 W for 10 s. Cross-sectional TEM images of Ni films after (e) 60 and (f) 100 cycles of ALE using a low plasma power of 100 W for 5 s.

As shown in Figures 2b and 3d, increasing the plasma source power to 300 W markedly reduced the RMS roughness of the Ni surface to 0.5 nm. This could be the consequence of a more uniform modified surface thanks to higher radical densities, increased plasma flux, or species formation. Therefore, the reactive intermediates adsorb more uniformly across both grain boundary and terrace sites, thereby equalizing local surface reactivity and suppressing site-selective etching.

### 3.2 ALE Behavior on Blanket Ni

The self-limiting nature of the ALE half-reactions is investigated using a N_2_/H_2_ plasma at 300 W, as this condition has proven to lead to efficient surface modification, followed by Hhfac exposure. EPC values were extracted from 10 ALE cycles performed in independent runs on fresh coupons for each condition. Figure 4a,b shows that the EPC as a function of the N_2_/H_2_ plasma exposure time saturates at 10 s with a value of 0.21 ± 0.02 nm/cycle. The EPC as a function of Hhfac dosing time showed saturation after 1.5 s (excluding the hold steps, as described in the Experimental Section). These results confirm the self-limiting nature of both half-reactions of the ALE process.

**Figure 4:**
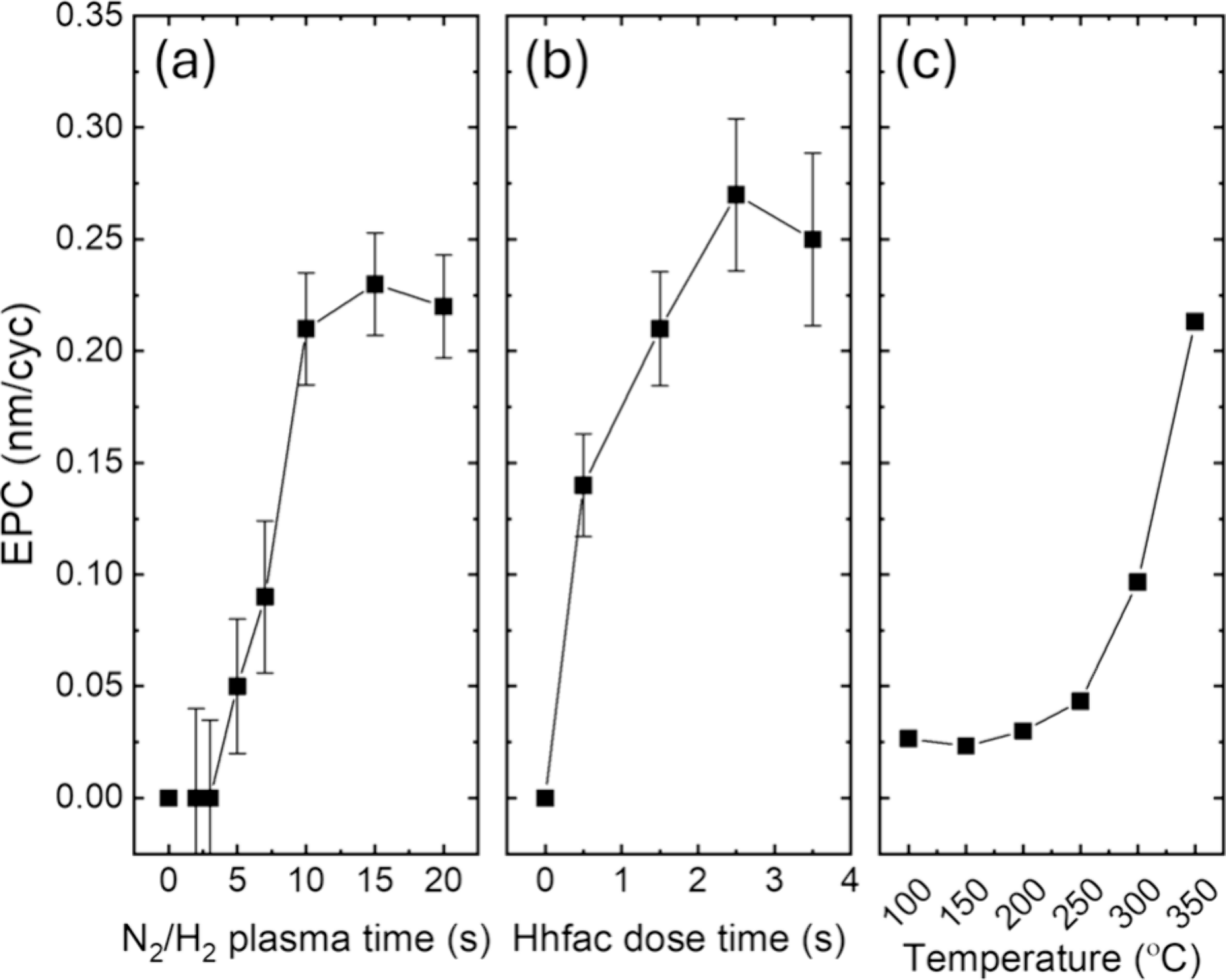
EPC as a function of (a) N_2_/H_2_ plasma exposure time for a fixed power of 300 W and a constant Hhfac dosing time of 1.5 s at 350 °C, (b) Hhfac dosing time at a fixed plasma power and time of 300 W for 10 s, respectively, operated at 350 °C as well, and (c) temperature at fixed plasma condition of 300 W for 10 s and Hhfac dosing time of 1.5 s. Fresh Ni samples were used for each EPC measurement.

Figure 4c displays the EPC of Ni as a function of the temperature. A notable increase begins around 250 °C, whereas below this threshold, the etch rate remains minimal (∼0.04 nm/cycle), indicating limited reactivity between Ni and Hhfac and low volatility of the Ni­(hfac)_2_ complex.^45^ Above 250 °C, enhanced thermal sublimation and increased volatility of Ni­(hfac)_2_ contribute to more efficient nickel removal, suggesting improved etch performance at elevated temperatures.

Figure 5i displays the Ni thickness as a function of the number of ALE cycles for different cyclic sequences, including: Hhfac only (red), N_2_/H_2_ plasma only (black), alternating cycles of N_2_/H_2_ plasma and Hhfac (blue), and the same, complemented by an O_2_ plasma step every 10 cycles (green). Repeating cycles of either only Hhfac dosing or only N_2_/H_2_ plasma exposure show negligible effects on the Ni thickness. After 10 cycles of Hhfac dosing steps, a thickness increase of approximately 0.5 nm is observed, likely due to the adsorption of Hhfac molecules on the Ni surface or the formation of nickel fluoride, as shown in Figure 1. In contrast, for alternating pulses of N_2_/H_2_ plasma and Hhfac (blue line), a consistent reduction in Ni thickness is observed. However, the etching process stalls after 10 cycles, likely caused by carbon residue buildup from Hhfac decomposition, as will be confirmed by XPS in Figure 6. A 15 s O_2_ plasma step was introduced after every 10 cycles of N_2_/H_2_ plasma and Hhfac, forming a (AB)_10_C super cycle, as shown in schematic Figure 5(ii). The green line demonstrates that the O_2_ plasma step, every 10 cycles, effectively removes these carbon-blocking residues, enabling continuous and linear etching. These results demonstrate that alternated dosing of N_2_/H_2_ plasma and Hhfac is required to achieve Ni etching, whereas no significant Ni etching is observed when only one of the two reactants is used. The thickness decrease of the Ni film for the full (AB)_10_C ALE process confirms the linearity of the etching process.

**Figure 5:**
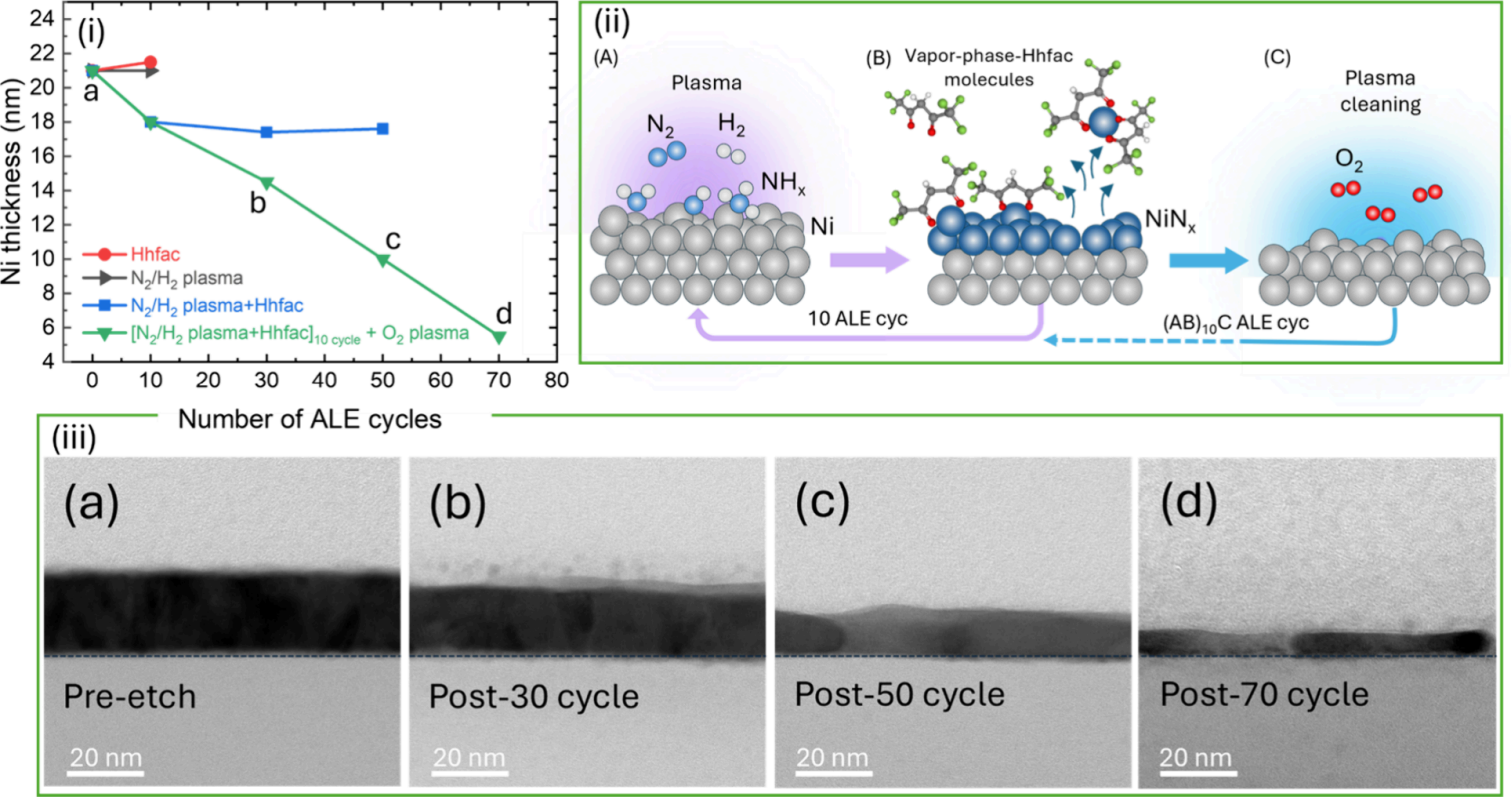
(i) Ni thickness as a function of ALE cycles for different process sequences. (ii) Reaction scheme of one full proposed ALE process flow, which shows a linear Ni thickness decrease (green line). (iii) Cross-sectional TEM images of Ni films (a) pre-etch and post (b) 30, (c) 50, and (d) 70 cycles of ALE using N_2_/H_2_ plasma and Hhfac followed by O_2_ plasma cleaning step every 10 cycles.

**Figure 6:**
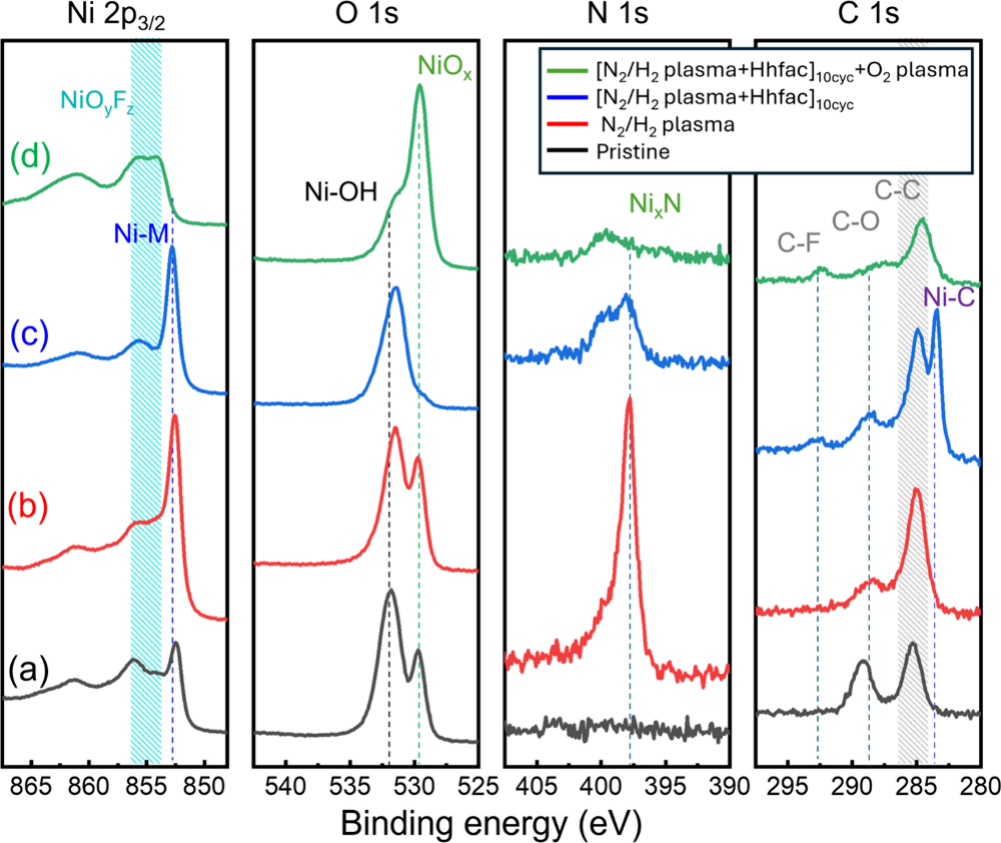
XPS detail scans of Ni 2p, O 1s, N 1s, and C 1s of (a) pristine Ni film as a reference, (b) after being treated by N_2_/H_2_ plasma showing intense N 1s peak, and (c) after being exposed to 10 ALE cycles ending with an Hhfac pulse, showing successful removal of Ni_*x*_N and residual of Ni–C on the surface, which was completely removed by 15 s of O_2_ plasma (d).

Cross-sectional TEM images of Ni films confirm the effectiveness of etching synergy before and after different numbers (n) of ALE full cycles [(AB)_10_C]_*n*_, as shown in Figure 5iii. The TEM images reveal a linear decrease in Ni thickness, reaching 5 nm after 70 cycles with minimal final RMS roughness. By comparing the thickness before and after ALE, the etch rate was calculated to be 0.21 nm/cycle, matching the EPC measured by *ex situ* XRR. This demonstrates the accurate etch control and the uniform nature of the proposed (AB)_10_C ALE process.

The surface composition was examined by XPS to identify the reaction products formed after each ALE half-cycle. As shown in Figure 6a, the Ni 2p_3/2_ spectrum of the pristine Ni surface exhibits a distinct metallic nickel peak (Ni-M), accompanied by a shoulder peak attributed to surface-related species like oxidation, fluorination, or nitridation (NiO_*y*_F_*z*_) and characteristic satellite features at binding energies of approximately 853, 855–858.5, and 860–864.5, respectively. Furthermore, the binding energy (BE) of O 1s and C 1s spectra of the pristine Ni 1s and C 1s shows peaks related to surface ambient oxidation (NiO_*x*_ at 529.6 eV and NiOH at 531.5 eV) and carbonation (C–C at 284.9 eV and C–OH at 289 eV).

After exposure to N_2_/H_2_ plasma, notable spectral changes were observed (Figure 6b). The intensity of the metallic Ni peak increased relative to oxide-related compounds, while the N 1s spectrum exhibits a distinct peak at a BE of 397.8 eV. This BE aligns with the typical range reported for metal nitrides (397–398 eV), suggesting the formation of a nickel nitride (Ni_*x*_N) phase on the surface. These spectral characteristics are consistent with those observed by Taylor et al.^11^ and Väyrynen et al.,^46^ who attributed the presence of the N 1s peak of metal nitride to the formation of the Ni_*x*_N compound. Similar spectra were observed for CVD Ni_*x*_N,^47^ and for ALD Ni deposited using a NH_3_ plasma,^48,49^ supporting the identification of the nitride phase as a product of the plasma exposure step.

Subsequent exposure of the Ni_*x*_N modified surface to Hhfac vapor was found to result in near-complete removal of the Ni_*x*_N layer. As shown in Figure 6c, the N 1s feature associated with Ni_*x*_N becomes barely detectable after Hhfac dosing, suggesting effective removal of NixN by Hhfac. In addition, the relative intensities of the NiO_*y*_F_*z*_ components in the Ni 2p_3/2_ spectrum are strongly reduced, suggesting the ability of Hhfac to remove also those compounds.^25,26^ The presence of a weak residual N 1s signal likely reflects incomplete reaction or subsaturation under the applied Hhfac dosing time (1.5 s), consistent with the slightly lower EPC observed under identical conditions (see the Supporting Information for a more detailed version of Figure 4).

XPS analysis also reveals an intense peak at 283.4 eV in the C 1s region, shown in Figure 6c, after 10 ALE cycles. This peak position corresponds to metal carbides,^50^ indicating the accumulation of carbon residues resulting from partial decomposition of the Hhfac precursor. In addition, a weak C–F peak is observed at a BE of 292.5, suggesting a minor residual of CF_*x*_ following Hhfac exposure. Such carbonaceous byproducts are known to inhibit surface reactions by blocking active sites, which are sufficient to block further etching progression (blue curve in Figure 5i). Similar behavior has been reported by Mameli et al. and Chittock et al., who observed that decomposition of organic etchants during ALE can lead to carbon-rich passivation layers that hinder further etching.^51,52^

To mitigate this effect, a 15 s O_2_ plasma step was introduced after every 10 cycles of N_2_/H_2_ plasma and Hhfac. As shown in Figure 6d, the metal-carbide peak completely disappeared after O_2_ plasma exposure, with only a minor residual of C–F remaining, indicating the effective removal of carbonaceous byproducts from the surface. In addition, a prominent NiO_*x*_ peak appears at a BE of 529.6 eV, consistent with the formation of nickel oxide, which can be easily etched by Hhfac. The incorporation of an O_2_ plasma cleaning step is crucial for maintaining effective Ni etching and preventing obstruction caused by carbon-based residues.

The crystallinity of Ni pre- and postetching was analyzed through grazing incidence X-ray diffraction (GIXRD) scans using an X-ray wavelength of 1.54 Å. The XRD results, displayed in Figure 7, reveal intense diffraction peaks at 44.5 and 51.8° (2θ), corresponding to the Ni (111) and Ni (200) lattice planes of a pristine 20 nm Ni film. The postetched Ni film exhibits identical peak positions and relative intensities as the pristine film, indicating that the etch process does not induce preferential etching or disrupt the crystal orientation of the grains.

**Figure 7:**
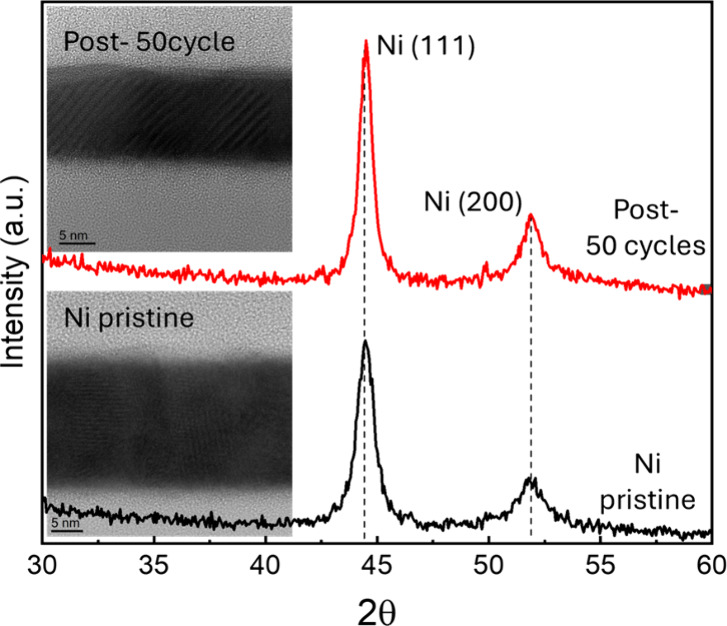
GIXRD of Ni film pre-etch and post 50 cycles of ALE using N_2_/H_2_ plasma and Hhfac followed by O_2_ plasma cleaning step every 10 cycles. The inset shows the related high-resolution TEM images.

A high-resolution cross-sectional TEM image is shown in the inset of Figure 8, for both pristine and postetched Ni film structures. The pristine film reveals a polycrystalline structure with distinct crystal grains and forms a closed, low-roughness layer approximately 20 nm thick. After 50 ALE cycles, the TEM image displays well-defined lattice fringes extending to the top surface layer, confirming that the Ni surface maintains its crystallinity following ALE. This outcome indicates that the ALE process does not induce significant damage or amorphization of the Ni film, effectively preserving its crystalline structure.

**Figure 8:**
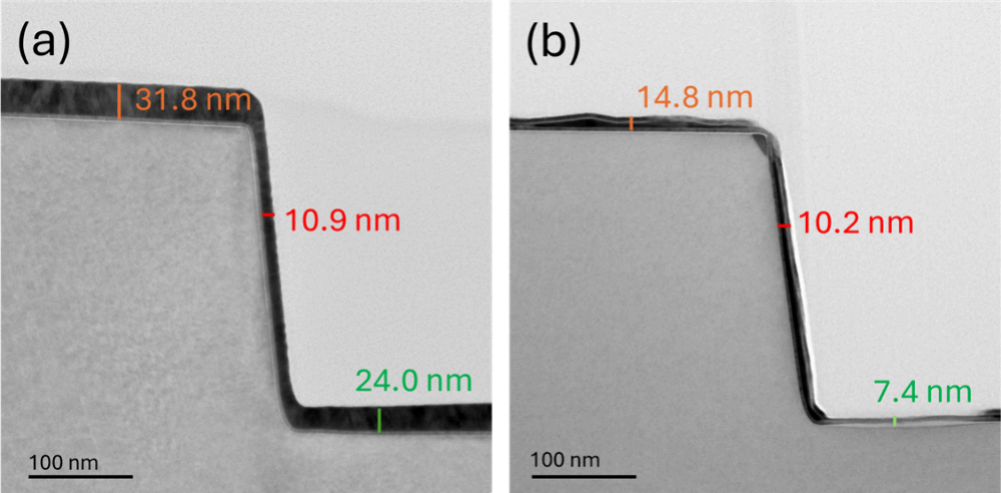
Cross-sectional TEM images of Ni film deposited on SiO_2_ step-like structure (a) before and (b) after 70 supercyclic (AB)_10_C ALE.

### 3.3 Anisotropic ALE of Ni

To capture the directional etching behavior of the proposed supercyclic (AB)_10_C ALE process, Ni films were deposited on prepatterned silicon, showing STI structures of fixed depth (300 nm) and variable horizontal dimensions. Figure 8a,b shows cross-sectional TEM images of a step structure in Si, before and after 70 cycles of the (AB)_10_C ALE process. The comparison reveals a notable difference in etch rates: the measured vertical EPC on top (orange) and bottom (green) of the step is around ∼0.24 nm, while the horizontal EPC is nearly zero. The Hhfac dosing step is isotropic by its nature. The strong contrast in etching directionality suggests that the N_2_/H_2_ plasma is responsible for the anisotropic etching observed here. The impact of the ion energy is, so far, difficult to estimate on the directionality of the process. This is due to technical challenges in performing ion energy measurements on this new system. It is highly possible that a modification of the plasma conditions (power, pressure, and gas flow) can modify the directionality of the ALE process. But this would require a dedicated study to understand the effect of the plasma conditions on the directionality of the ALE process. Overall, our result demonstrates that the conditions of our ALE process not only ensure controlled, layer-by-layer removal of Ni but also maintain the integrity of sidewalls and neighboring structures. This is of particular advantage for advanced semiconductor and nanofabrication technologies.

## 4 Conclusions

A novel anisotropic plasma-assisted ALE process for Ni was demonstrated by using alternating exposures to N_2_/H_2_ plasma and Hhfac, revealing the self-limiting and controllable nature of both half-reactions. The etch per cycle (EPC) exhibits a strong dependence on plasma composition, power, and substrate temperature. Optimal etching was achieved at an H_2_/(H_2_ + N_2_) of 0.67, where the supposed combination of radical densities and NH_*x*_ generated species is leading to the higher EPC, i.e., leading to efficient surface activation.

XPS analysis confirmed the formation of a transient Ni_*x*_N layer during the N_2_/H_2_ plasma step, which is subsequently removed upon Hhfac exposure, completing the ALE cycle. The near disappearance of the Ni_*x*_N-related N 1s feature after Hhfac dosing validates the self-limiting nature of both the nitridation and ligand-removal reactions. At low plasma power, preferential etching along grain boundaries leads to increased surface roughness, whereas higher plasma powers equalize surface reactivity and yield smoother etched surfaces.

Importantly, the O_2_ plasma intermediate cleaning was introduced after every 10 ALE cycles to counteract the buildup of carbonaceous residues originating from Hhfac decomposition. This additional cleaning step effectively removed metal–carbide species detected near 283.4 eV (C 1s), restored the active Ni surface, and enabled continuous linear etching.

Structural characterization by GIXRD and HRTEM confirmed that the ALE process preserves both crystallinity and surface planarity with no evidence of lattice distortion or amorphization. Furthermore, the etching exhibited anisotropic directionality, favoring vertical over lateral removal, suggesting an essential role of the ions in the surface activation during the N_2_/H_2_ plasma step.

Overall, this work establishes a robust and chemically controlled ALE strategy for Ni, integrating N_2_/H_2_–Hhfac cyclic chemistry with O_2_ plasma cleaning. Unlike traditional ion milling, this approach leaves no Ni-based residues or damage, and it offers indications of achievable anisotropy. The process still has drawbacks (relatively high temperature and low etch rate), but targeted engineering and further process optimization can improve performance. We believe that our process will pave the way for simple, residue-free, damage-free, and anisotropic metal etching that is crucial for integrating advanced low-resistance interconnects.

## Supplementary Material



## Notes

Additional details for Figure 4, including overdose conditions for both N_2_/H_2_ plasma and Hhfac; RMS roughness values for each data point; AFM image at high Hhfac flow (PDF)
