# Supplementary material for: Atomic Layer Etching of Nickel Using N2/H2 Plasma Exposure and Hexafluoroacetylacetone
Source: ACS Appl Electron Mater. 2026 Apr 24;8(9):3834–42. doi: 10.1021/acsaelm.5c02655 (PMC13175136; doi:10.1021/acsaelm.5c02655)
Supplement: Supplementary file 1 [file el5c02655_si_001.pdf]

Supporting Information

Title:

Atomic layer etching of nickel using N_2/H_2 plasma exposure and hexafluoro-acetylacetone

Authors:

Ali Mohamed Ali[1,2]*, Guillaume Krieger[3], Jean-Philippe Soulié[2], Harm C. M. Knoops[3,4], Wilhelmus M. M. Kessels[3], Stefan De Gendt[1,2], Souvik Kundu[2], Jean-François de Marneffe[2]

Affiliation:

[1]Dept. of Chemistry, K.U. Leuven, Celestijnenlaan 200F, B-3001 Leuven, Belgium.
[2]imec v.z.w., Kapeldreef 75, B-3001 Leuven, Belgium.
[3]Dept. of Applied Physics, Eindhoven University of Technology, P.O. Box 513, 5600 MB Eindhoven, Netherlands.
[4]Oxford Instruments Plasma Technology, Govier Way, Bristol BS35 4GG, United Kingdom.

Corresponding authors:

Tel: * + 32484242581

E-mail: * ali.abdelgawad@imec.be

The overexposure or overdose condition for both N_2/H_2 plasma and Hhfac are shown in figure (see below). Figure (b) shows how the overdose of Hhfac increases the surface roughness. Looking in more details, shorter N_2/H_2 plasma or longer exposure to Hhfac results in roughening of the surface of the Ni after etch. The plasma-induced roughness is closely tied to the plasma power and exposure duration, as highlighted by the results shown in Figure 2(b) in

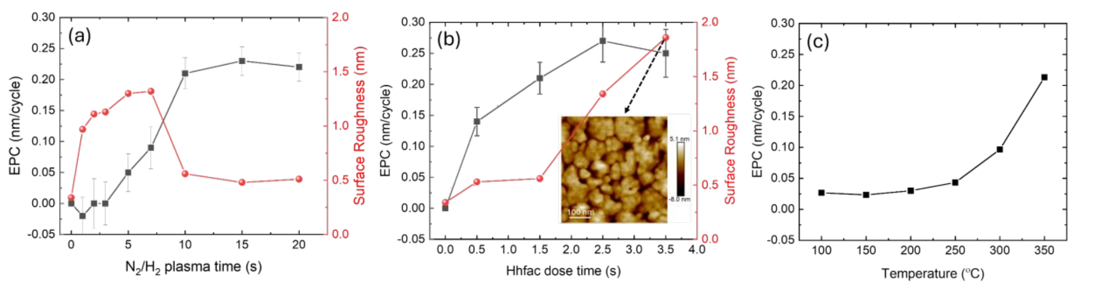

EPC (black) and surface roughness (red) as a function of (a) N_2/H_2 plasma exposure time for a fixed power of 300 W and constant Hhfac dosing time of 1.5 s, (b) Hhfac dosing time at a fixed plasma power and exposure time of 300 W and 10 s, respectively. The inset shows an AFM scan of a 5 × 5 μm^2 region of Ni etched with a longer Hhfac dosing time of 3.5 s. and (c) temperature.

the manuscript. On the other hand, extending the Hhfac dosing beyond 1.5 sec led to an increased surface roughness, as shown in the inset of the attached figure, suggesting that prolonged exposure caused the Hhfac to attack grain boundaries underneath, which had been modified due to diffusion of N and H radicals. Therefore, the Hhfac exposure was maintained at 1.5 sec to balance effective etching and minimal roughening.
